# Inflammatory Pathways to Carcinogenesis: Deciphering the Rheumatoid Arthritis–Lung Cancer Connection

**DOI:** 10.3390/cancers17081330

**Published:** 2025-04-15

**Authors:** Boushra Abou Hjeily, Briana Candace Nevaneeth, Włodzimierz Samborski, Zoltán Szekanecz, Bogna Grygiel-Górniak

**Affiliations:** 1 Rheumatology Research Group, Department of Rheumatology, Rehabilitation and Internal Diseases, Poznan University of Medical Science, 61-701 Poznan, Poland; 2 Department of Rheumatology, Rehabilitation and Internal Diseases, Poznan University of Medical Science, 61-701 Poznan, Poland; wlodzimierz.samborski@ump.edu.pl; 3 Division of Rheumatology, Faculty of Medicine, University of Debrecen, 4032 Debrecen, Hungary; szekanecz.zoltan@med.unideb.hu

**Keywords:** rheumatoid arthritis, lung cancer, iatrogenic effect of DMARDs, lung cancer risk factors

## Abstract

Rheumatoid arthritis (RA) is the most common chronic autoimmune arthropathy. If the disease is aggressive or left untreated, it becomes debilitating, affects a patient’s functionality, and reduces the quality of life. Disease-modifying anti-rheumatic drugs (DMARDs), both conventional, targeted, and biological, decrease the disease progression and are key components of effective treatment. Recently, there has been a continuous debate about the possible carcinogenicity of various DMARDs. Lung cancer is a leading cause of cancer death worldwide. The available data show an increased risk of lung cancer in RA patients, but the link between RA and cancer is poorly understood. Carcinogenesis in RA seems to be related to chronic inflammation, familial predisposition, risky behaviors (e.g., smoking), and iatrogenic complications. The main mechanisms of carcinogenic processes in patients with RA are the up-regulation of interleukin-6 (IL-6) cytokine production and wingless/integrated WNT signaling. Up-regulation of WNT5A is an important mechanism that links chronic inflammatory pathways to carcinogenesis observed in RA patients. Concomitant up-regulation of transcription factor STAT3 promotes cell proliferation and inhibits apoptosis. Conversely, suppressed inflammatory processes by DMARDs may decrease the risk of lung cancer. In this article, we discuss the molecular mechanisms of lung cancer in RA and the role of DMARDs in this process. Furthermore, we analyze the molecular effect of drug-induced cancer, which affects transcription factors and thus modulates carcinogenic processes. Finally, we describe risk factors and present preventive and therapeutic approaches.

## Simple Summary

Lung cancer is a well-known long-term complication of rheumatoid arthritis (RA). However, the management of lung cancer concerning RA has been poorly studied and managed without a focused set of guidelines. In this extensive review, we have discussed the carcinogenic impact of using disease-modifying anti-rheumatic drugs (DMARDs), the molecular processes involved in the carcinogenic mechanisms of DMARDs, the factors involved in the development of lung cancer in RA patients, and the prevention and treatment of lung cancer associated with RA. Future long-term studies must be conducted to establish guidelines on the screening and treatment of cancers in RA patients. It should also focus on setting guidelines for the prevention of lung cancer in RA patients on long-term DMARD therapy, as well as determine the carcinogenic potential of new DMARDs on the market.

## 1. Introduction

Rheumatoid arthritis (RA) is a chronic autoimmune disorder that affects the joints and surrounding tissues. It is characterized by inflammation, pain, and stiffness, and, if left untreated, it can lead to progressive joint damage and disability. Various factors, such as genetics, environment, and lifestyle, influence this disease [1]. The inflammation associated with this disease can cause changes in the lung tissue, making it more susceptible to excessive cell proliferation typical of the neoplastic process [2]. One of them is lung cancer—a malignant tumor that is the leading cause of cancer death worldwide and is primarily caused by exposure to tobacco smoke [3]. The two most common types of lung cancer are small-cell lung cancer and non-small-cell lung cancer [4].

RA patients are often treated with immunosuppressive drugs, which, when taken for many years (usually decades), can also contribute to the development of lung cancer [5]. These data are mainly based on oncological and hematological registers; however, immunosuppressants are used in much higher doses in these diseases than in rheumatic diseases. Many molecular pathways have been described during the use of disease-modifying anti-rheumatic drugs (DMARDs); however, there are still many questions about their iatrogenic features. Without a doubt, these drugs have ushered in a new era in rheumatology. They prevent disease progression and the development of severe complications in RA patients.

On the other hand, DMARDs, by suppressing the immune response, can also stimulate carcinogenic processes. Unfortunately, the link between RA and the lung’s carcinogenic process is poorly understood. Still, recent data suggest that individuals with RA may be at an increased risk of developing lung cancer. There is also evidence that RA smokers are at an even greater risk of lung carcinogenesis than individuals who do not have RA. This has been attributed to the synergistic effects of smoking and inflammation typical for RA on the lungs [6].

Despite the association between RA and lung cancer, the relationship between these two conditions is not well established, particularly since progression to cancer is mainly driven by persistent inflammation. During carcinogenesis, tumor-associated antigens (TAAs) with adhesive properties are present on tumor cells and can be expressed by inflammatory leukocytes. In addition, soluble TAAs are elevated in many rheumatic diseases. The described mechanisms demonstrate possible overlapping pathogenetic pathways in rheumatic and neoplastic processes [7].

Therefore, this review describes the relationship between RA and lung cancer, including the molecular mechanism of cancer development and the possible pathomechanism of drug-induced lung carcinogenesis in patients with rheumatoid arthritis. Understanding the relationship between RA and lung cancer is important for developing targeted interventions to reduce the risk of lung cancer in RA patients.

## 2. Materials and Methods

**Figure 1:**
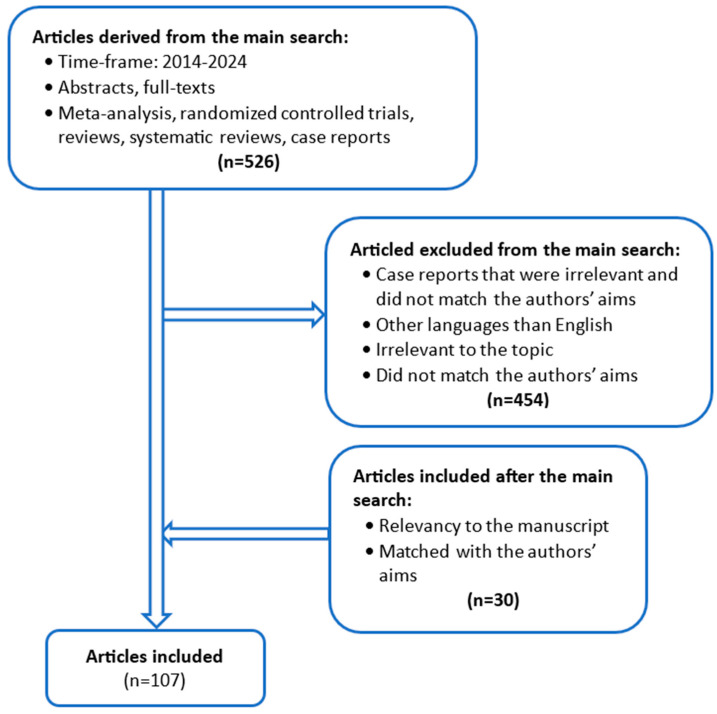
Literature search and methodology.

This review article is formed from a PubMed and Google Scholar literature search that included meta-analysis, case reports, case studies, systematic reviews, randomized control trials (RCTs), and prospective and retrospective cohorts. Out of these, a stark emphasis was placed on systematic reviews, meta-analyses, and cohorts, while individual case studies, case reports, and RCTs were mainly referred to. The search words used to include manuscripts were lung cancer, rheumatoid arthritis, cancer risk, cancer, methotrexate, leflunomide, sulfasalazine, biologic DMARDs, JAK inhibitors, hydroxychloroquine, adalimumab, certolizumab, anakinra, etanercept, golimumab, infliximab, tocilizumab, sarilumab, rituximab, and abatacept. Conjunction words such as AND and OR were also used to maximize search further. From the manuscripts, only English manuscripts were included. The literature search included articles from a timeframe of 2014 and onwards. Other relevant articles aligned with the authors’ aims for the review were included outside the established timeframe to include key information. The search criteria are shown in Figure 1.

## 3. Epidemiology

Lung cancer is the leading cause of cancer-related deaths (17.6% of all cancer deaths) [8]. According to GLOBOCAN 2020, an estimated 2.2 million new lung cancer cases and 1.8 million lung cancer-related deaths occurred in 2020 [9]. In 2021, lung cancer was the second most commonly diagnosed cancer in men and women [10]. Since 1985, the incidence of lung cancer has increased by 51% [8]. These data are surprising, especially since carcinogenic processes are more prevalent in men than women (119,100 vs. 116,660 estimated new cases, respectively) [10]. Furthermore, the incidence of lung cancer is higher in developed than in developing countries due to the increase in smoking (676,681 vs. 672,221 cases, respectively) [11]. Besides this, malignant lung lesions develop more frequently in older people. Most newly diagnosed cases occur in patients aged 55 to 74 years (53% of cancer cases), followed by those over 75 years of age [12].

Nearly 90% of lung cancers are linked to smoking [13]. Smoking is the most important risk factor for developing lung cancer, contributing to 80% of lung cancer deaths in men and 50% in women. Lung cancer has several histopathological types [12,13]. The most common type is adenocarcinoma (38% of all lung cancer cases). Lower prevalences are typical for squamous cell carcinoma (20%), small cell carcinoma (14%), and large cell carcinoma (3%) [14].

Unfortunately, a higher risk of carcinogenesis is observed in patients with autoimmune diseases. For example, RA patients are at an increased risk of developing several cancers: leukemia, non-Hodgkin’s lymphoma, and lung cancer. In a study conducted on a Spanish population with RA from 1999 to 2005, it was found that lung cancer had the highest IR per 10,000 (95% CI) of 31 (15 to 65) among all other diagnosed cancers [15]. This type of cancer is 43% more common in patients with RA than in the general population and is characterized by higher mortality rates [16].

A meta-analysis by Simon et al. showed an overall increase in malignancy in RA patients but also reported that there was a decrease in the risk of breast and colorectal malignancies compared with the general population. However, the reasons behind the increased risk of lung cancer are not fully understood and can be attributed to a modifiable risk factor such as smoking. Smoking is also a risk factor for developing RA itself. Still, it is currently unknown whether smoking is a direct cause of lung cancer in RA patients or if it is related to inflammatory pathways typical for RA [17,18]. Moreover, the severity of RA, through failed therapy or prolonged disease course in a patient, can also contribute to the heightened risk of lung cancer. Not effective treatment of RA patients causes chronic inflammation in pulmonary tissue, which contributes to uncontrolled neo- and carcinogenesis [17,19,20,21,22,23,24,25,26,27,28,29,30,31,32,33,34,35,36].

## 4. Molecular Mechanism of Lung Cancer Development in RA Patients

The complex pathway of cancer development in RA patients involves changes in B-cell activation. One of the cytokines associated with B-cell stimulation and induction of IgG expression is interleukin-6 (IL-6). This cytokine is produced by monocytes and macrophages at sites of acute inflammation, as well as by fibroblast-like synoviocytes (FLSs) [19]. IL-6 contributes to inflammatory diseases by activating a signaling pathway that involves the protein JAK1 (Janus kinase 1). JAK1 triggers the phosphorylation of the transcription factor STAT3. Once phosphorylated, STAT3 forms dimers and translocates to the nucleus, where it initiates the transcription of various genes that promote cell proliferation and inhibit apoptosis [37].

The JAK-STAT pathway can be a double-edged sword. On one hand, this pathway contributes to oncogenesis in various cancers, including lung cancer, by promoting cell proliferation, survival, angiogenesis, and immune evasion [38]. Inhibition of this pathway has been shown to protect against tumor development and progression. On the other hand, JAK inhibitors also suppress the immune system, which could theoretically reduce its ability to fight cancer cells (this mechanism is used in the treatment of RA by JAK inhibitors) [39]. Furthermore, there is a concern that by interfering with immune surveillance, JAK inhibitors could potentially increase the risk of malignancies [39], although the current data equivocal [40].

Notably, this signaling cascade leads to the up-regulation of WNT5A, which induces the canonical WNT signaling pathway. Wnt5a activates several non-canonical pathways independently of β-catenin’s transcriptional activity. These include the planar cell polarity (PCP) pathway and the Wnt/Ca^2+^ pathway, which play roles in cell polarity, migration, adhesion, and fundamental processes in cancer progression and inflammation [41]. Overexpression of WNT5A up-regulation has been observed in various cancers, including lung cancer [42]. Interaction between tumor cells and stromal cells leads to WNT5A up-regulation in the early stages of primary tumors, promoting the development and spread of metastasis [43]. The feedback loop between STAT3 and WNT5A signaling underscores a molecular bridge connecting chronic inflammation to carcinogenesis. In RA, chronic inflammation is a hallmark of the disease, characterized by persistent STAT3 activation, which could contribute to the aberrant activation of WNT5A signaling. This mechanism not only drives the pathological inflammation seen in RA but also establishes a pro-tumorigenic environment, potentially linking RA to an increased risk of developing certain types of cancer, including lung cancer [41].

Growing evidence has implicated Wnt5a in chronic inflammatory disorders [41]. Enhanced canonical WNT signaling in chronic inflammation in RA results in elevated fibronectin levels and enhanced fibroblast invasiveness through proMMP-3 [44]. The increased survival of fibroblast-like synoviocytes (FLSs) leads to an elevated production of WNT and downstream effectors, including IL-6 [44]. In the context of lung cancer and inflammation in RA, the Wnt5a signaling pathway seems to be a potential molecular link, given its involvement in both oncogenic processes and chronic inflammation [42]. Wnt5a-related mechanisms in regulating cancer cell behavior and their effects on inflammatory processes make Wnt5a a candidate for shared molecular pathways between lung cancer and RA.

In conclusion, research into examining the molecular mechanisms of lung cancer in RA patients is few. Due to the extensive factors involved in chronic inflammation that drive the disease processes of RA and in oncogenesis, it is necessary to examine all molecular pathways to establish certainty behind the molecular cause of the heightened incidence of lung cancer in RA. For future research, it is also important to study the molecular effects of JAK inhibitors on the JAK-STAT pathway to note whether interfering with immune surveillance can cause malignancies in RA patients. Currently, research into JAK inhibitors is limited and new as the drugs were approved for use in recent years [17,18,19].

## 5. Drug-Induced Lung Cancer in RA Patients

Recent data show the effectiveness of RA treatment and decrease in the risk of RA complications due to the everyday use of synthetic disease-modifying anti-rheumatic drugs (DMARDs), including synthetic, conventional medications such as methotrexate—MTX, leflunomide—LEF, sulfasalazine—SN, chloroquine—CQ, hydroxychloroquine—HCQ, and target DMARD, including Janus kinase (JAK) inhibitors (e.g., tofacitinib, baricitinib, and upadacitinib). Besides this, biological DMARDs are used, including anti-cytokine drugs (adalimumab, anakinra, certolizumab, etanercept, golimumab, infliximab, tocilizumab, and sarilumab) and non-anti-cytokine medications such as abatacept and rituximab [45]. Such treatment increases the effectiveness of RA treatment and improves therapeutic effects, but, simultaneously, some drugs are associated with the stimulation of cancerogenic processes, particularly during long-term use (summarized in Table 1).

Balancing between benefits and the risk of developing cancer is an important clinical problem. However, the lack of proper RA treatment increases inflammatory processes, which can also raise the risk of cancer. It should also be emphasized that most data on the pro-carcinogenic effects of drugs have been described in oncological patients who are given much higher doses of immunosuppressive medications than in rheumatic patients. For example, in oncology, methotrexate is used in high doses (HDMTX), which means a dose of 500 mg/m^2^ i.v. or higher [46,47]. HDMTX is used for various cancers, e.g., acute lymphoblastic leukemia, osteosarcoma, and lymphomas (both in children and adults) [46,48,49]. The recommended dose of MTX in RA is much smaller and ranges from 25 to 30 mg/weekly (starting from an initial dose of 10–15 mg/weekly), which explains a much lower risk of adverse effects, including carcinogenesis [45]. If the patient responds well to the therapy, small doses of MTX are given prolongedly, usually for many years. Hence, data on the risk of developing cancer in CTD should be interpreted with great caution while emphasizing the beneficial effect of these drugs in reducing autoimmune processes obtained with the use of their small drug doses.

**Table 1:** The risk of lung cancer during various DMARD therapy in RA patients.

Lung Cancer Risk in RA Patients During Synthetic and Biological Drug Therapy					
Synthetic DMARD	**Medication**	**Drug**	**Analyzed Group with RA**	**Number of Patients with Lung Cancer/Calculated Risk**	**Ref. Number**
	conventional	Methotrexate(MTX)	*n* = 459 Follow-up: 1983–1998	*n* = 14 (0.03%) SIR (95% CI) 2.9 (1.6–4.8)	[21]
		Leflunomide(LF)	*n* = 14 patients: 12 patients with RA 1 patient with LORA 1 patient with JIA Type of therapy: a combination of LEF and JAKi Study duration: June 2017–March 2022	*n* = 1 Type of cancer: non-small cell lung cancer	[50]
		Sulfasalazine (SFN)	N/A	N/A	N/A
		Chloroquine (CQ)/Hydroxychloroquine (HCQ)	*n* = 100,000 adult patients Follow-up: 6.7 in non-HCQ users and 7.82 years in HCQ users.	*n* = 15 (0.00015%) non-HCQ users’ cHR (95% CI) 1.128 (0.54–2.34); *p* = 0.7457 HCQ users’ aHR (95% CI) 1.019 (0.48–2.16); *p* = 0.9616	[51]
	target	Tofacitinib (TOFA)	*n* = 4362 age: ≥50 years with ≥1 additional cardiovascular risk factor Dose: 5 mg or 10 mg twice a day Follow-up: 6 years (2014–2020)	*n* = 30 (0.007%) IR (95% CI): 0.28 (0.19 to 0.39) *n* = 24 NSLC (0.005%) IR (95% CI) 0.22 (0.14 to 0.33) *n* = 5 SCLC (0.001%) IR (95% CI) 0.05 (0.01 to 0.11)	[52]
		Baricitinib (BARI)	*n* = 3770 RA patients Study duration: 9.3 years	*n* = 26 (respiratory and mediastinal neoplasms malignant and unspecified) EAIR (95% CI)- 0.17 (0.11 to 0.25)	[53]
		Upadacitinib (UPA)	*n* = 1629 RA patients who were on MTX regimen previously *n* = 877/1629 RA patients using UPA + background MTX therapy, including patients who switched from placebo or ADA (from 1629 RA patients) Study duration: 3 years	*n* = 3 (patients who switched to UPA from placebo or ADA developed unspecified lung cancer stage IV) IR N/A	[54]
biologic	anti-cytokine	Adalimumab(ADA)	*n* = 1629 RA patients using MTX *n* = 277/1629 RA patients using ADA + background MTX therapy, including patients who switched from UPA Study duration: 3 years	*n* = 2 1 patient diagnosed with SCC of the lung stage IV 1 patient switched from upadacitinib to adalimumab developed large cell lung cancer IR N/A	[54]
		Anakinra	*n* = 1346 RA adult patients in total *n* = 1116/1346 RA patients in anakinra-treated group Study duration: 3 years	*n* = 1 patient (type of cancer N/A) SIR (95% CI)- 0.31 (0.01 to 1.75) SIR (95% CI)- 0.31 (0.01 to 1.75)	[55]
		Certolizumab(CERTO)	*n* = 975 Age: ≥18 years; Study duration up to 12 months from drug initiation	*n* = 2 patients (type of cancer N/A) 0.24 IR/100 patient-years (95% CI) (0.06 to 0.94)	[56]
		Etanercept(ETA)	*n* = 558 early RA patients *n* = 714 long-standing RA patients age: ≥18 years study duration: >15 years	*n* = 6 early RA patient group *n* = 8 long-standing RA patient IR N/A	[57]
		Golimumab (GLM)	*n* = 530 GLM s.c. and 157 GLM-i.v. patients Mean age: 55.8- 57.7 years Study duration: 14 years	*n* = 2 patients 0.19 rate/100 patient-years	[58]
		Infliximab(IFX)	*n* = 11,767 RA patients without prior diagnosis of cancer *n* = 3457 infliximab-treated patients Study duration: 2001–2011	*n* = 25 IR per 10,000 patient-years (95% CI) = 20 (13 to 30)	[59]
		Whole-group anti-TNFα inhibitors	Adding TNFα inhibitors to sDMARD does not alter the cancer risk in RA patients *n* = 11,767 RA using anti-TNFα vs. *n* = 3249 RA patients treated with non-biologic (synthetic) DMARDs	Comparable risk of cancer HR 0.83 (95% CI 0.64 to 1.07) in both groups	[59]
		TNF inhibitors (ADA, CERTO, ETA, GLM, IFX)	Swedish registry RA patients (*n* = 69 308) Time of observation: 2001–2018	Not increased risk of the overall relative risk of cancer (HR = 1.0) when compared to RA patients not treated with bDMARD and tcDMARD	[60]
		Tocilizumab	*n* = 69,308 RA patients *n* = 2895 tocilizumab-treated RA patients Study duration: 6.5 years	aHR (95% CI)- 1.3 (0.8 to 2.2) HRb (95% CI)- 1.2 (0.7 to 2.0)	[60]
		Sarilumab	N/A	N/A	N/A
	non-anti-cytokine	Abatacept	*n* = 4134 age: <20 to ≥75 years old	*n* = 13 patients IR (95% CI)- 0.15 (0.08 to 0.27)	[61]
		Rituximab	*n* = 409,706—RA patients exposed to RTX since 2006 and from RA clinical trials with an 11-year follow-up. Study duration: 2006–2017	*n* = 164 *n* = 87; malignant lung neoplasm *n* = 27; pulmonary adenocarcinoma *n* = 20; metastatic lung cancer *n* = 19; SCC *n* = 11; bronchial carcinoma IR N/A	[62]

aHR—adjusted hazard ratio; cHR—crude hazard ratio; CI—confidence interval; CIR—crude incidence ratio; EAIR—exposure-adjusted incidence rate; GLM—golimumab; GLM-IV—golimumab intravenous; HCQ—hydroxychloroquine; IR—incidence ratio; I.v.—intravenously; JAKi—Janus kinase inhibitor inhibitors; JIA—juvenile idiopathic arthritis; LORA—late-onset rheumatoid arthritis; MTX—methotrexate; N/A—not available or not reported; NSLC—non-small lung cancer; RA—rheumatoid arthritis; RTX—rituximab; s.c.—subcutaneously; SCC—squamous cell carcinoma; SCLC—small cell lung cancer; SIR—standardized incidence ratio; tsDMARD—targeted synthetic DMARD.

### 5.1. Methotrexate

Methotrexate (MTX) is one of the DMARDs used as a first-line treatment in patients with various rheumatic diseases. In RA, MTX inhibits the disease’s activity and protects the joints from destruction. This medication is effective and has a good safety profile [22]. However, after analyzing its metabolic pathways and adverse effects, we see that MTX shows some pro-carcinogenic activity.

**Figure 2:**
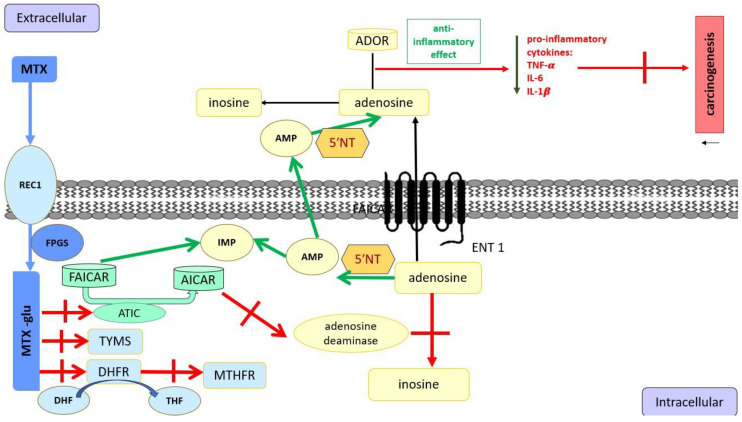
Mode of Action of Methotrexate: Methotrexate (MTX) is absorbed into cells via RFC1 and forms its active form, MTX-glu, via FPGS. MTX-glucan inhibits AICAR transformylase. Accumulated intracellularly, AICAR inhibits adenosine deaminase and AMP deaminase. As a result, the inhibition of irreversible degradation of adenosine is observed. AMP is extracellularly converted to adenosine by the ecto-5′-nucleotidase (5′NT). MTX-glu can inhibit DHFR, MTHFR, TYMS, and ATIC, causing the inhibition of the formation of purines and pyrimidines and decreasing the proliferation of inflammatory cells. Extracellularly, adenosine binds to ADOR, which has an anti-inflammatory effect and decreases the synthesis of pro-inflammatory cytokines IL-1β, TNF-α, and IL-6. FAICAR: 5-form-aminoimidazole-4-carboxamide ribonucleotide; AICAR: 5-aminoimidazole-4-carboxamide ribonucleotide; ADOR: 4 G-protein coupled receptors; cAMP: cyclic adenosine monophosphate; DHF: dihydrofolate; DHFR: dihydrofolate reductase; ENT 1: equilibrative nucleoside transporter 1; IL-6: interleukin-6; IL-1β: interleukin-1β; IFN-γ: interferon-gamma; MTHFR, methylenetetrahydrofolate reductase; TYMS: thymidylate synthetase; THF: tetrahydrofolate; TNF-α: tumor necrosis factor-alpha. On the other hand, adenosine can bind to 4 G-protein coupled receptors (ADOR), making it a paracrine signaling molecule. ADORA2A and ADORA3 are two of these receptors that have anti-inflammatory effects, decreasing the production of neutrophil superoxide and the pro-inflammatory cytokines IL-1β, TNF-α, and IL-6 [22,23]. Mentioned cytokines participate in an inflammatory state and are also detected in carcinogenesis.

Many hypotheses were published to explain the methotrexate mechanism’s influence on carcinogenic processes. For example, MTX influences the adenosine signaling pathway, increasing its release [22]. MTX blocks 5-aminoimidazole-4-carboxamide ribonucleotide (AICAR) transformylase (ATIC), increasing intracellular AICAR (Figure 2). AICAR blocks adenosine deaminase, inhibiting the transformation of adenosine to inosine. Adenosine is transported by equilibrative nucleoside transporter 1 (ENT1) to extracellular space, increasing extracellular adenosine. Extracellular adenosine has an anti-inflammatory effect and increases cAMP, inhibiting pro-inflammatory cytokines such as TNF-α, IFN-γ, and IL-1β [22].

**Figure 3:**
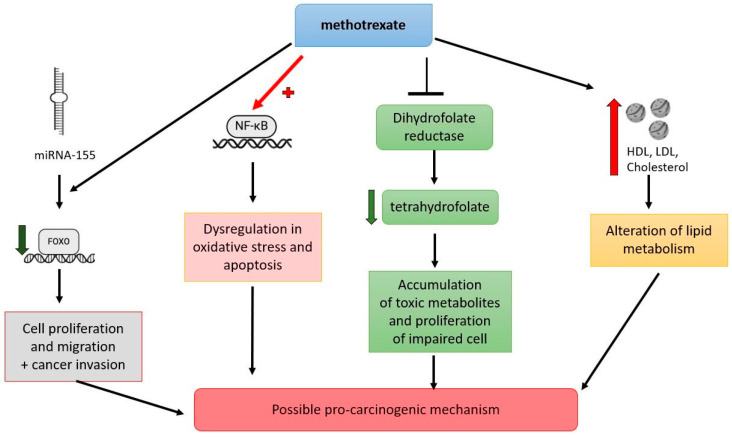
Pro-carcinogenic Mechanism of Methotrexate: miRNA155: micro RNA 155; FOXO: forkhead box O; NF-kB: nuclear factor kappa B; HDL: high-density lipoproteins; LDL: low-density lipoproteins.

Besides the adenosine signaling pathway, MTX influences the expression of microRNA-155 (miR-155), a small non-coding RNA that plays a critical role in various cellular processes, including inflammation, immune response, and cancer (Figure 3). miR-155 targets FOXO3a, a member of the forkhead box O (FOXO) family of transcription factors, known for its tumor suppressor functions such as inducing apoptosis, cell cycle arrest, and DNA repair. The interaction between miR-155 and FOXO3a mediated by MTX leads to decreased FOXO3a levels, promoting cell proliferation, migration, and invasion in cancer, thus contributing to the tumor’s aggressive behavior [24]. The activation of NF-κB, a key regulator of immune response and inflammation, is another mechanism by which MTX may exert pro-carcinogenic effects. In nephrotoxicity, methotrexate-induced activation of NF-κB leads to oxidative stress and apoptosis, processes that can contribute to carcinogenesis if dysregulated. This pathway suggests that MTX’s impact on inflammation and oxidative stress has broader implications for cancer development, particularly in tissues sensitive to these processes [25].

MTX interferes with folate metabolism by inhibiting dihydrofolate reductase and thus reduces the availability of tetrahydrofolate, which is necessary for synthesizing purines and thymidylate. This inhibition affects DNA synthesis and repair, accumulating toxic metabolites and impairing cell proliferation. While these effects are leveraged therapeutically in cancer treatment, they can also be a potential pathway through which methotrexate might contribute to carcinogenic processes, particularly in the context of long-term use or in combination with other risk factors [26].

MTX may indirectly facilitate cancer progression by altering lipid metabolism, a critical process for tumor growth. Cancer cells rely heavily on de novo lipogenesis and lipid uptake for membrane biosynthesis, energy storage, and signaling. Ronda et al. reported that a 6-month methotrexate treatment in patients with RA led to a significant increase: 8% in total cholesterol, 9% in low-density lipoprotein (LDL) cholesterol, and 15% in high-density lipoprotein (HDL) cholesterol [63]. Similarly, Rodriguez-Jimenez et al. observed comparable changes in patients with RA treated with MTX for 24 weeks, noting increases of 11% in total cholesterol, 9% in LDL cholesterol, and 6% in HDL cholesterol [64]. Navarro-Millán et al. documented a more pronounced increase of 30% in total cholesterol, 28% in LDL cholesterol, and 39% in HDL cholesterol in a larger cohort of RA patients (*n* = 226) following 24 weeks of methotrexate treatment [65]. Methotrexate-induced changes in lipid profiles could inadvertently enhance lipid scavenging by cancer cells, which often overexpress LDL receptors or exploit HDL as a cholesterol source [66]. These lipids are essential for sustaining rapid proliferation, metastasis, and oncogenic signaling via lipid-derived molecules like prostaglandins and oxysterols [67]. Research has demonstrated that certain cancer cells overexpress scavenger receptor class B type 1 (SR-B1), facilitating the uptake of HDL cholesterol, which in turn supports tumor growth and proliferation. It was indicated that SR-B1 is overexpressed in lung cancer cells. A study analyzing lung adenocarcinoma tissues found that SR-B1 expression was significantly higher in cancerous tissues (96%) than adjacent normal lung tissues (56%), suggesting a role in tumor aggressiveness and poor prognosis [68]. Additionally, studies have shown that cancer cells, including those in lung cancer, may exploit HDL and its components to meet their increased cholesterol demands for membrane biosynthesis and signaling, thereby promoting malignancy [69].

The carcinogenic potential of MTX was identified nearly three decades ago. Despite this, its significant clinical benefits in many connective tissue diseases with good drug toleration have largely mitigated concerns regarding its association with cancer [70].

A study by Buchbinder et al. showed a direct effect of MTX in increasing lung cancer risk in RA patients (*n* = 459). The study was based on 4145 person-years of follow-up (average 9.3 years), and 87 malignancies were detected (melanoma, non-Hodgkin’s lymphoma, and lung cancer). The authors showed that the estimated 50% excess risk of malignancy among methotrexate-exposed RA patients relative to the general population (SIR 1.5, 95% confidence interval [95% CI]) ranges from 1.2 to 1.9, with an almost 3-fold increase in lung cancer (SIR 2.9, 95% CI 1.6–4.8) [21]. A similar risk of lung cancer in RA patients was described by Smitten et al. and was estimated at 1.63 (1.43–1.87) [20]. The data of Simon et al. showed that the prevalence of malignancies in RA patients is a two-fold increase in lung cancer compared to the general population, particularly in patients treated with MTX compared to biologic DMARDs, predominantly TNF inhibitors [17]. In patients with RA, the standardized incidence ratios SIRs (95% CI) for lung cancer ranged from 1.36 to 2.9, and the total pooled SIR (95% CI) was 1.64 (1.51–1.79) [17]. This can be partially related to the higher prevalence of lung cancer in general populations, which is one of the leading causes of cancer-related deaths, achieving about 17.6% worldwide [8]. Moreover, an inflammatory state typical for RA can be related to an increased risk of malignancies compared with the general population (Table 1).

A more extensive study by Solomon et al. compared cancer-associated risk with MTX to other non-biologic and biologic DMARDs and reported 30 out of 2866 cases of cancer in RA patients who received MTX, where only two of these cases were lung cancer [71]. The cessation of MTX therapy, described in a case report, showed a beneficial effect on carcinogenic processes in a 72-year-old man with RA being on long-term MTX and prednisolone therapy. The patient was diagnosed with squamous cell carcinoma in the left bronchus and a concurrent B-cell lymphoma-type lymphoproliferative disorder in the right lung. After discontinuing MTX, a newly detected nodule in the left lower lung resolved, suggesting MTX’s role in contributing to lymphoproliferative lesions alongside lung cancer [72].

In conclusion, analysis of MTX pathways has shown some pro-carcinogenic potential, which may influence the development of lung cancer in patients with RA. On the other hand, high disease activity and persistent inflammation in patients treated inadequately or ineffectively (especially in those in whom MTX was not used or is contraindicated) may stimulate carcinogenic processes. Hence, further studies are needed to analyze the incidence of lung cancer in the context of assessing RA activity. Understanding the long-term effects of methotrexate use in patients with RA may help to identify patients with a high risk of developing cancer. Mainly, stopping smoking is of crucial importance because it not only affects the development of lung cancer but is also an additional factor that increases the activity of RA [4,73]. Appropriate monitoring of patients may enable rapid diagnosis and effective treatment not only of RA but also rapid detection of new lung lesions. Since MTX is the first-line drug in RA patients, it should be used according to EULAR/ACR recommendations in every person with this disease after assessing the possible risk of lung cancer.

### 5.2. Leflunomide

Leflunomide (LEF) is another DMARD commonly used in various rheumatic diseases. LEF active metabolite (A77-1726) is an inhibitor of dihydroorotate dehydrogenase—a mitochondrial enzyme, which participates in de novo pyrimidine ribonucleotide uridine monophosphate (rUMP) synthesis [74]. LEF, at low doses, may promote cancer cell survival and proliferation by transiently increasing extracellular signal-regulated kinase (ERK) phosphorylation, decreasing p38 mitogen-activated protein kinase (MAPK) and c-Jun N-terminal kinase (JNK) phosphorylation, and activating Akt signaling, which inhibits pro-apoptotic pathways and enhances anti-apoptotic gene transcription. In contrast, high doses of LEF can induce mitochondrial proliferation in human osteosarcoma cells and rat-liver-derived cells [28,29].

LEF treatment is usually initiated in patients with RA after the lack of methotrexate effectiveness or the development of MTX-induced side effects. One of the complications of LEF, but not very common, is the development of interstitial lung disease. A Japanese study showed that 61 RA patients who received LEF out of 5084 (1.2%) developed interstitial lung disease (ILD) [75]. The study reported that lung disease typically develops within 20 weeks of initiating LEF treatment, although the timing varies due to multiple factors. Specifically, patients who received a loading dose and those with pre-existing ILD tended to present earlier, within the first 12 weeks [75].

Pre-existing ILD and pneumonitis caused by MTX are recognized as risk factors for pulmonary disease after LEF implementation. Consequently, using LEF as a substitute for MTX is restricted under these conditions [76]. A case study of a LEF-treated patient shows a direct relation between LEF initiation treatment and lung injury in patients with a history of RA-related ILD and previous therapy with MTX. After treatment with LEF, the patient developed rapid progression of ILD, leading to severe lung injury [77]. A similar case report describes a patient (previously treated with MTX) who showed the development of pulmonary nodules while being treated with LEF. The pulmonary nodules disappeared after six months of LEF discontinuation, showing the potential role of this drug in their development [78].

A case series of five-year observations of 14 patients with inflammatory arthritis, including 12 patients with RA, one patient with late-onset rheumatoid arthritis, and one patient with juvenile idiopathic arthritis (JIA) who were initiated on combination therapy of LEF and a Janus kinase inhibitor reported that such a treatment was effective. They demonstrated a favorable safety profile across most patients. However, one patient discontinued LEF and the JAK inhibitors therapy after the new onset of non-small cell lung cancer (NSCLC), which had developed with a history of 25 pack years of smoking [50]. This particular case raised concerns about the potential link between the combination of LEF and JAK inhibitors and the development of lung malignancies, especially in patients with predisposing factors such as smoking. The description of the single case reports, case series, and single studies show some pro-carcinogenic potential of this medication. Still, the benefits of it in RA patients outweigh the potential side effects. Nevertheless, future analyses in large groups are required to show which of the predisposing factors (RA activity, LEF, JAKi, or smoking) has the most potential influence on lung cancer development.

### 5.3. Sulfasalazine

Sulfasalazine is another DMARD commonly used in RA and other inflammatory diseases, mainly ulcerative colitis [79]. Sulfasalazine and its metabolites, mesalazine, and sulfapyridine are anti-inflammatory drugs. They block the synthesis of various inflammatory molecules, including prostaglandins and leukotrienes, by inhibiting cyclooxygenase 1 and 2 [79]. In RA patients, sulfasalazine can be used in combined treatment with MTX and hydroxychloroquine when MTX alone does not produce the desired effects [80]. Sulfasalazine rarely causes lung injury, but it can present pulmonary symptoms such as cough, dyspnea, and chest pain [81]. However, also severe complications such as pulmonary edema and eosinophilic or interstitial pneumonia are reported [82].

The data of Parry et al. showed that among the patients who reported adverse effects of sulfasalazine, the majority suffered from respiratory symptoms (80% had dyspnea, 64% had a cough, 90% had hypoxia, and 92% had pulmonary infiltrates). Most commonly, interstitial pneumonitis was detected (22% developed eosinophilic pneumonia, 12% pulmonary hypersensitivity, 12% fibrosing alveolitis, and 8% lung granulomas)—however, 90% of patients improved after drug cessation. Besides the aforementioned pulmonary disorders, no studies or reported cases of sulfasalazine-induced lung cancer have been published till now [81].

### 5.4. Antimalarial Medications

Antimalarial medications, such as chloroquine (CQ) or hydroxychloroquine (HCQ), are conventional DMARDs widely used to treat various connective tissue diseases, including RA [83]. HCQ’s mode of action in inhibiting autophagy can become a source of cancer progression. Late autophagy inhibition of cancer cells can eventually lead to their cell survival. HCQ also impacts MHC-1 antigen presentation and processing, leading to an escape of cancer cells from the immunosurveillance of the immune system. It has also been found that HCQ induces the phosphorylation of c-Jun, a substrate needed for JNK (c-Jun N-terminal kinases) signaling. An increase in JNK signaling is required for NF-κB activation and p62 expression.

Additionally, at a 25 μm HCQ dose added to melanoma cells, HCQ causes an accumulation of p62 protein that leads to the induction of NF-κB activation and further p62 protein translation. This leads to the expression of cell survival genes and eventual resistance to cell apoptosis. Similarly, it was noted that at lower doses of HCQ, the drug increased the mRNA and protein levels of HIF-1ɑ and IL-8 in melanoma and squamous cell carcinoma cells. HIF-1ɑ is a known contributor to skin cancer progression, angiogenesis, and increased tumor survival. Lastly, HCQ has also been shown to cause an increase in anti-apoptotic factors such as Bcl-2 and Bcl-XL through NF-κB activation. This leads to an overall anti-apoptotic effect and cancer cell survival and progression [84,85]. Thus, despite in vitro data describing the impact of hydroxychloroquine on the potential risk of developing lung cancer, no clinical data demonstrate an association between this drug use and the development of cancer in patients with RA.

### 5.5. JAK Inhibitors

JAK inhibitors are a well-known group of drugs used in various diseases ranging from rheumatic to dermatologic and gastroenterological diseases. They primarily work through numerous JAK families, such as JAK1, JAK2, JAK2, and TYK2. The three drugs that are routinely used and discussed in rheumatic diseases are tofacitinib (TOFA; works on JAK families in this order- JAK3 > JAK2 > JAK1), baricitinib (BARI; JAK1, JAK2, and TYK2), and upadacitinib (UPA; JAK1 > JAK2 and JAK3) [86].

Recent evidence has raised concerns about the use of JAK inhibitors due to an observed increase in malignancy risk, particularly for lung cancer. Preclinical studies provide additional insight into the mechanisms by which JAK inhibitors may contribute to cancer progression. Research by Shimaoka et al. demonstrated that cytokine signaling inhibitors, including JAK inhibitors, can enhance cancer metastasis by depleting natural killer (NK) cells, especially in lung tissues. In an experimental mouse model of colon cancer, JAK inhibitors significantly reduced NK-cell counts, thereby impairing the immune response against lung metastatic cells [87]. This NK-cell depletion mechanism may partially explain the elevated lung cancer risk observed in clinical studies.

Clinical data confirmed the ratio between cancer risk and JAKi treatment. For example, the study of Fleischmann et al. showed that the long-term safety and efficacy of upadacitinib vs. adalimumab over 3 years were similar (a long-term extension of the SELECT-COMPARE trial, a randomized, controlled phase 3 trial in patients with active RA and an inadequate response to MTX). The rates of malignancy, excluding non-melanoma skin cancer (NMSC), were similar between upadacitinib and adalimumab during 156 weeks of follow-up. Malignancies in patients receiving upadacitinib included malignant melanoma (*n* = 3), lung cancer (*n* = 3), and breast cancer (*n* = 2); malignancies in patients receiving adalimumab comprised of colorectal cancer (*n* = 3) and lung cancer (*n* = 2). The observation was short, and thus, further studies, including larger groups and longer observations, are needed [54].

A comprehensive meta-analysis by Russell et al. evaluated malignancy risks associated with JAK inhibitors across various diseases and found a significant association with lung cancer. This study reported an estimated hazard ratio (HR) of 1.48 for cancer risk in patients treated with JAK inhibitors compared to non-JAK therapies, with a notably higher incidence of lung cancer specifically [88]. These findings are echoed by the ORAL Surveillance trial, which compared TOFA with TNF inhibitors (adalimumab at a dose of 40 mg every 2 weeks or etanercept at a dose of 50 mg once weekly) in patients with RA and no history of cancer. Patients in this study were 50 years or older and had at least one additional cardiovascular risk factor. All patients were randomly assigned in a 1:1:1 ratio to receive TOFA 5 mg twice daily (*n* = 1455) or 10 mg (*n* = 1456) twice daily or a TNF inhibitor (*n* = 1451). The median follow-up time was four years. Incidence rates (IRs; patients with first events/100 patient-years) and HRs were calculated for adjudicated malignancies excluding non-melanoma skin cancer (NMSC), NMSC, and subtypes. This randomized controlled trial found that patients receiving TOFA had a 1.4-times higher risk of developing overall malignancies, including a substantially increased risk of lung cancer (such relation was not observed in patients before 50 years old; the risk increased over the age of 65). Specifically, the lung cancer incidence rate in the TOFA group was 0.7 per 100 patient-years, compared to 0.5 per 100 patient-years in the TNF inhibitor group [52].

Similarly, based on the ORAL Surveillance trial, Ytterberg et al. published an analysis that compared the risk of major adverse cardiovascular events (MACEs) and malignancy (confirmed non-melanoma skin cancer) in a group of patients with RA treated with TOFA or a tumor necrosis factor (TNF) inhibitor. During this time, the incidence of malignancy was higher with the combined TOFA dose (4.2%; *n* = 122 patients) than with the TNF inhibitor (2.9%; *n* = 42 patients). The hazard ratios for malignancy were 1.48 (95% CI, 1.04 to 2.09). However, the efficacy of both drugs (TOFA and anti-TNF) was similar across study arms. Interestingly, the differences in the risks of MACE and malignancy between TOFA and the TNF inhibitor were greater in patients aged 65 years and older than in younger patients. Because the rates of all-cause death (including cancer) and pulmonary embolism were higher with TOFA 10 mg twice daily than with the TNF inhibitor, the dose of this drug was reduced to 5 mg twice daily during the study. This study showed a higher risk of malignancy with TOFA than with anti-TNF alpha agents. Still, it was not proven whether this risk was specific to patients only with TOFA or if it also applies to other JAK inhibitors [89].

The recommendations of the Task Force of EULAR recommend the cautious use of JAK inhibitors in patients with cancer and only without alternative treatment methods (no reliable data confirming the indications for treatment with these drugs in cancer patients) [90]. Unfortunately, there are few studies (as mentioned above) describing the effect of these drugs [52,89]. Therefore, the EULAR recommendations include consideration of risk factors for cardiovascular events and malignancies before prescribing a JAK inhibitor, such as age > 65 years, current or past smoking history, cardiovascular and thromboembolic events, and risk factors for malignancy (current or past history of malignancy, except for successfully treated non-melanoma skin cancer) [45]. Similarly, experts of the Task Force believe that JAK inhibitors can be used in such patients only if there are no suitable alternative methods of treatment available [90].

## 6. Biologic Therapy

Biological DMARDs are a group of drugs commonly used in RA. Each DMARD works differently, but all inhibit inflammatory reactions through different mechanisms, for example, inhibiting the release of pro-inflammatory cytokines (TNF, IL-1, or IL-6) [91]. The main classes of biological DMARDs include TNF inhibitors (adalimumab, etanercept, golimumab, certolizumab, and infliximab), IL-1 inhibitors (anakinra), IL-6 inhibitors (Tocilizumab), and T-cell inhibitors (abatacept). All biological DMARDs are proteins that target-specific receptors or molecules involved in inflammation [92].

Clinical data provide evidence that biological DMARDs increase the risk of infections (including bronchitis and pneumonia) and may reactivate latent tuberculosis. Biological DMARDs also cause other pulmonary disorders, including interstitial lung disease, pulmonary hypertension, and bronchiectasis. The study of Joseph et al. showed that 111 of 332 patients treated with biological DMARDs developed some pulmonary disorders (29 patients had interstitial lung disease, and 76 patients had airway disease encompassing conditions such as thickening of the bronchial walls, bronchiectasis, bronchiolitis, air trapping, and atelectasis (partial or complete lung collapse)) [93]. The risk of malignancy in RA patients taking biological and synthetic DMARDs shows synthetic DMARDs (no specific DMARDs were mentioned by authors) slightly increase (about 1.15-fold) the risk of cancer development (mainly lung and blood). Patients receiving biological DMARDs did not have an increased risk of developing cancer [5].

One of the biological anti-cytokine medications is anakinra (anti-IL1), used in Still disease (a systemic form of RA). This medication does not appear to increase the risk of cancer. However, it should be used with caution in RA patients with a history of malignancy [55].

Another biological anti-cytokine medication is tocilizumab (anti-IL6). In a cohort analysis of various biologic and targeted synthetic DMARDs on lung cancer risk, tocilizumab exhibited a crude incidence rate of 1.6 per 1000 person-years (19 cases in 11,812 person-years), leading to an HR of 1.3 (95% CI 0.8–2.2) [60].

In the same study, abatacept showed a relatively lower rate of 0.9 per 1000 person-years, with 13 cases in 13,988.1 person-years and an HR of 0.7 (95% CI 0.4–1.3) [60]. Similarly, other data suggested that the risk of lung cancer in patients using abatacept is low. Still, this drug may increase the risk of malignancy, especially non-melanoma skin cancer (NMSC) [94,95]. A recent study by Long et al. indicates that the depletion of CD8 T cells may be associated with remission of RA and an increased risk of malignancy. Abatacept has a probable, indirect influence on CD8 T-cell depletion, which results in reduced RA symptoms. However, CD8 T-cell depletion may also stimulate carcinogenic pathways in cells and cause aggressive cancers [96].

The observational studies of abatacept analyzed by the Task Force of EULAR show an increase in cancer incidence in patients treated with this drug compared with other targeted therapies (including tocilizumab and rituximab) in patients with RA and no history of cancer. The adverse pro-cancer effects of abatacept may result from the characteristics of the drug, which has a mechanism of action opposite to that of ipilimumab and other immune checkpoint inhibitors (ICIs). Therefore, abatacept should be used in patients with cancer cautiously and only without alternative treatment methods (no reliable data confirming the indications for treatment with these drugs in cancer patients) [90].

### TNFα Inhibitors

**Figure 4:**
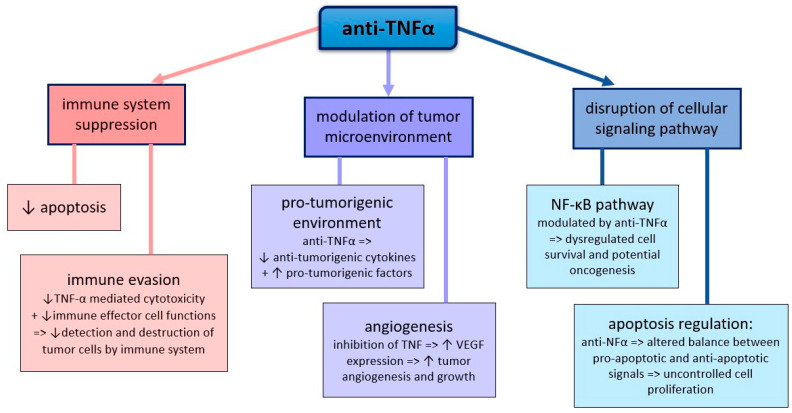
Potential Carcinogenic Mechanism of TNFα: TNFα—tumor necrosis factor-alpha; NF-kB—nuclear factor kappa B; VEGF—vascular endothelial growth factor.

Adalimumab, etanercept, golimumab, certolizumab, and infliximab inhibit TNFα activity. TNFα is an inflammatory cytokine involved in multiple immune pathways (Figure 4). TNFα plays a key role in host immunosurveillance of tumors, mediating the destruction of cancer cells through apoptosis and promoting immune recognition of aberrant cells [97,98]. Anti-TNFα therapies impair this surveillance, allowing newly nascent tumor cells to evade immune detection and elimination [30]. First, this process may be due to inhibition of TNFα-mediated cytotoxicity against tumor cells or suppression of immune effector cell function [30]. Secondly, TNFα affects the tumor microenvironment by regulating inflammation, angiogenesis, and cell proliferation [31,32].

Anti-TNFα therapy may inadvertently promote a tumor-promoting microenvironment by reducing the synthesis of anti-tumorigenic cytokines and increasing the expression of pro-tumorigenic factors [33,34]. TNFα blockade stimulates vascular endothelial growth factor (VEGF) expression, facilitating angiogenesis and tumor growth 29. TNFα also interacts with various cell signaling pathways that regulate cell survival, proliferation, and death. Inhibition of TNFα can disrupt these pathways, leading to uncontrolled cell proliferation and survival. Moreover, anti-TNFα therapy might modulate the NF-κB pathway, which is involved in cell survival, and its dysregulation increases cancer risk [35]. Also, TNFα blockade may affect the balance between pro-apoptotic and anti-apoptotic signals in cells, potentially allowing cancer cells to proliferate [36]. Therefore, recent data on the pro-carcinogenic effects of anti-TNFα therapy are conflicting. It is hypothesized that inhibition of the immune system by reducing apoptosis by anti-TNFα may promote carcinogenic processes in the long term [99,100,101]. The mechanisms by which anti-TNFα agents may contribute to carcinogenesis are multifaceted and include changes in immune function, modulation of the tumor microenvironment, and effects on cell signaling pathways [102,103,104].

Lung cancer is particularly significant in RA due to the interplay between chronic lung inflammation, smoking, and immunosuppressive therapies, with some reports indicating up to 1.36 times increased risk in lung cancer compared to the general population [60,105].

A comparable prevalence of lung cancer was described in RA patients treated with various anti-TNFα therapies. For example, the study of Weinblatt et al., lasting over 15 years, described lung cancer development in 14 RA patients treated with etanercept out of the group 558 patients with early RA (*n* = 6) and 714 with chronic disease (*n* = 8) [57]. Lung cancer was also reported during golimumab treatment; however, the risk of its development is very low. A 14-year observational study included 530 patients using subcutaneous and 157 intravenous golimumab revealed cancer in two patients, which corresponded with the incidence rate of 0.19 per 100 patient-years [58]. Similar lung cancer prevalence was observed during certolizumab treatment (in two from 975 adult patients, giving the incidence rate (IR) of 0.24 per 100 patient-years (95% CI: 0.06 to 0.94; study duration of up to 12 months)) [56].

A large study from the British Society for Rheumatology Biologics Register (BSRBR) analyzing 11,767 RA patients without prior cancer who received TNF inhibitors found that the rates of solid cancers are comparable to those in 3249 patients without prior cancer treated with non-biologic (synthetic) DMARD HR 0.83 (95% CI 0.64 to 1.07). The unadjusted hazard ratios (HRs) for TNF inhibitors were 0.57 (95% CI 0.40–0.82) for all drugs from this group. Moreover, there was no difference in the relative cancer risk for any of the individual TNF inhibitor drugs [the risk was 0.64 (95% CI 0.42–0.98) for etanercept, 0.59 (95% CI 0.36–0.97) for infliximab, and 0.49 (95% CI 0.29–0.76) for adalimumab] [59]. Similarly, data from the Swedish registry show patients with RA (*n* = 69 308) treated with TNF inhibitors (adalimumab, certolizumab, etanercept, golimumab, and infliximab) or other biologic and targeted synthetic DMARDs (ABA, RTX, BARI, TOFA, and tocilizumab) within 2001–2018 had not increase cancer risk when compared to RA patients not treated with biologic and targeted synthetic DMARDs. The overall relative risk of cancer with TNF inhibitor (HR = 1.0) did not increase. It did not change with time since the treatment started, the duration of active treatment, or attained age compared to patients not treated with biologic and targeted synthetic DMARDs [60]. Nevertheless, among patients receiving TNF inhibitors, those with a history of lung disease or smoking had a potentially heightened risk of lung cancer when compared to those on non-biologic DMARDs [17,59].

Recently published recommendations highlighted that in patients with a history of solid cancer (excluding melanoma) who require targeted anti-rheumatic therapy, TNF inhibitors may be preferred over other treatment options based on the best available evidence. Published data have not shown an increased risk of new cancer with TNF inhibitors compared with conventional synthetic DMARDs (csDMARD) in patients with a history of solid cancer. Therefore, the Task Force of EULAR unanimously preferred TNF inhibitors over other treatment options, mainly due to the abundant data available for these drugs and the paucity of translational data on other therapeutic options, including RTX and IL6, IL-12/23, IL-17, and IL-23 inhibitors [90].

Given the above data, TNF inhibitors are a safe treatment option in patients with arthritis and cancer. However, a comprehensive patient evaluation should include disease duration, time from anti-TNFα implementation, and additional risk factors for lung cancer (e.g., smoking or COPD). Such monitoring should allow early detection of lung cancer and improve surveillance for the potential carcinogenic effects of these medications. Regular clinical monitoring and adherence to clinical guidelines for cancer screening remain paramount in managing patients receiving long-term anti-TNFα therapy.

Interesting evidence was provided by a systematic review of the literature by Sebbag et al., who analyzed 14 studies to provide a relative measure of the risk in patients with inflammatory arthritis with a history of cancer receiving targeted therapy (including b/tsDMARDs) versus csDMARDs (*n* = 4428 patients with new or recurrent cancer, median follow-up from initiation of treatment 4.52 years). The patients analyzed mainly were patients with RA, most often with solid tumors, and most often treated with TNF-alpha inhibitors. The authors showed that targeted therapies were not associated with an increased risk of cancer recurrence compared with csDMARDs (overall hazard ratio for cancer recurrence 0.92; 95% CI 0.74 to 1.15). Although the study did not specifically consider lung cancer but mainly mixed cancer or breast cancer, it showed that there was no significant difference between the treatments analyzed in the occurrence of new cancer, even in patients treated less than 5 years after cancer diagnosis [106].

## 7. Treatment of Lung Cancer in RA Patients

**Figure 5:**
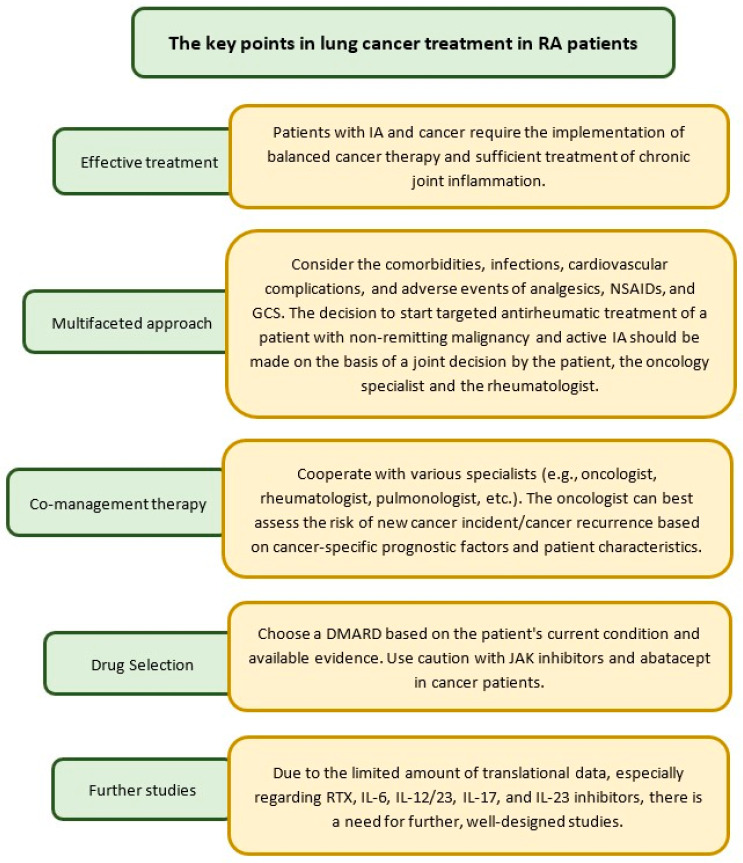
The treatment recommendation in patients with cancer and arthritis; IA—inflammatory arthritis; NSAIDs—non-steroidal anti-inflammatory arthritis; GCS—glucocorticosteroids.

The treatment of lung cancer should be based on the histopathological type, stage, and duration of the tumor. The diagnosis of lung cancer in patients with RA should always be associated with effective treatment of both the tumor and the rheumatic disease itself (Figure 5). Furthermore, patients starting treatment should be informed about possible methods of RA treatment and, in the case of cancer, also about anti-cancer therapy with all its potential complications. Regular monitoring of musculoskeletal symptoms and cancer progression allows for appropriate adjustment of treatment at every stage of the disease. A multidisciplinary approach is recommended, allowing for coordinated health by oncologists, therapists, pharmacists, physiotherapists, dieticians, and social care specialists [107].

Recently, EULAR published recommendations for the initiation of targeted therapies in patients with inflammatory arthritis (IA) and a history of cancer. These guidelines emphasize that effective treatment of IA is necessary in patients with a history of cancer because chronic inflammation may increase the risk of some cancers (e.g., lymphomas). At the same time, the risk of cancer relapse associated with targeted antirheumatic therapy must be balanced against the risk of insufficient treatment of chronic inflammation. Experts emphasize the need for a multifaceted approach, which should consider the assessment of co-morbidities, infections, cardiovascular complications, and adverse events of analgesics, NSAIDs, and corticosteroids used during ineffective DMARD treatment [90].

A key point in co-management of the therapy of a patient with cancer and arthritis is assessing the benefits and risks at the time of initiation of targeted therapy in patients with a history of cancer. Therefore, a rheumatologist should cooperate with cancer organ specialists such as oncologists, hematologists, pulmonologists, etc. [108,109]. Moreover, cancer in remission should not delay the initiation of targeted antirheumatic treatment, especially in active arthritis [110,111]. Previous studies have shown that if antirheumatic treatment was initiated within 5 years of cancer diagnosis, there was no significant difference in the risk of new cancer development between targeted therapies (anti-TNF) and csDMARDs [90].

If immune checkpoint inhibitors (ICIs) are indicated for lung cancer, patients with low-active or inactive RA should also be monitored by a rheumatologist. In contrast, high-risk patients with active RA must receive ICIs only on a case-by-case basis, and immunosuppressive regimens must be adjusted based on tolerability. In addition, both groups of patients should be closely monitored for RA flares and drug toxicity [112]. Due to the lack of specific recommendations for lung cancer treatment in patients with RA, further studies are required to develop standardized treatment guidelines [107].

## 8. Prevention of Lung Cancer in RA Patients Treated with Conventional DMARDs and Biological DMARDs

Current recommendations consider active cancer to be a general contraindication to DMARD use due to increased infection rates [56,78]. Nevertheless, according to EULAR recommendations, a baseline immunosuppressive regimen should be kept in RA patients after the development of lung cancer at the lowest dose possible (for GCS, below 10 mg prednisone per day if possible). However, many patients may have an RA flare or immune-related adverse events, which require the use of GCS and/or DMARDs [113]. Therefore, conventional synthetic DMARDs should be considered individually and in consultation with an oncologist [107]. Moreover, pre-existing autoimmune rheumatic and/or systemic diseases should not preclude the use of cancer immunotherapy [113].

Cancer risk increases if chronic inflammation is typical for RA, which is not adequately treated. Therefore, it is essential to emphasize that effective RA treatment reduces local and systemic inflammation and, thus, indirectly diminishes the risk of carcinogenesis. Thus, if RA develops in patients with lung cancer, initially, local and/or systemic glucocorticoids (GCSs) should be considered to reduce rheumatic symptoms; if the goal is achieved (low RA disease activity), systemic GCS should be tapered to the lowest effective dose to control the symptoms. However, if the disease is still active, csDMARDs should be considered in patients with insufficient response to acceptable doses of GCS or requiring GCS-sparing. Some of the csDMARDs, such as MTX, CspA, azathioprine, and LEF, have been associated with some risk of malignancy development. Still, it has not been proven that they increase lung cancer specifically. More data are needed to determine whether this association is due to a specific drug, and such analysis should include a large group of patients using specific monotherapy or combined treatment during long-time observation [18].

In severe rheumatic disease with systemic symptoms or with insufficient response to csDMARD, bDMARD may be considered. Among them, TNFα or IL-6 inhibitors are the preferred options for inflammatory arthritis [113]. Since rituximab has good clinical outcomes; it is used widely [18]. In the case of a lymphoma diagnosis, RTX therapy may be preferred over other treatment options because of the potential for beneficial B-cell depletion. In patients with non-remitted malignancy not in remission and active IA, initiating targeted antirheumatic therapy should be based on a shared decision between the patient, the cancer specialist, and the rheumatologist [90].

In patients with RA, personal and family history of cancer should always be considered. When initiating RA treatment (particularly in patients with a personal or family history of lung cancer in the past), it is recommended to educate patients about any signs of cancer (e.g., unintentional weight loss, dyspnea, prolonged cough, and hemoptysis). If such symptoms develop, a quick diagnosis and evaluation should be made. The quick diagnosis increases the possibility of immediate treatment, diminishing the risk of cancer progression and severe complications. However, based on expert opinion, current recommendations do not specify pulmonary symptoms or time for screening [107,112]. Therefore, general recommendations should be followed to prevent further risk of lung cancer. Smoking cessation can reduce the risk of lung cancer and have a positive effect on RA activity [4,73]. In every case of RA and cancer disease, the shared decision with the patient should be made considering the future rheumatic and oncologic therapy. The decision to hold or continue the cancer immunotherapy should be based on the severity of rheumatic immune-related adverse events, the extent of the required immunosuppressive regimen, the tumor response and duration, and the future oncology treatment plan in a shared decision with the patient [113].

In each RA patient, it is important to assess the risks of using different DMARDs to ensure optimal and safe treatment for RA patients, especially those at potential risk for lung cancer. When selecting a drug, it is important to avoid undertreating patients who could benefit from different treatment options. The choice of drug, the goal of treatment, costs, and patient preferences should always be discussed with the patient.

## 9. Conclusions

Since every year more patients are faced with the diagnosis of tumors, the risk of cancer development in connective tissue diseases is increasing problem. Lung cancer is considered the leading cause of cancer death worldwide. This type of neoplasm can develop in patients with RA. Therefore, it is crucial to understand and determine the potential risk of lung cancer in RA subjects. There are many risk factors associated with the occurrence of lung cancer in RA. Some are modifiable, such as smoking cessation, effective anti-rheumatic therapy, or adequate nutrition. In contrast, others, such as a genetic predisposition to RA, the disease duration, or a family history of lung cancer, are inherited between generations. Smoking status (current or former) is a significant risk factor in RA patients because, in combination with certain medications (such as TNFα inhibitors), it can increase the risk of cancer. In addition, chronic lung changes in progressive RA also increase the risk of carcinogenesis. Therefore, in case of any lung problems in RA patients, preventive examinations (e.g., regular X-rays or CT scans) are a priority.

In addition, a multidisciplinary approach to the treatment of RA patients with lung cancer is required to prevent RA exacerbations that may complicate lung cancer treatment. The interactions between chemotherapeutic drugs and DMARD should be considered to avoid and/or minimize iatrogenic effects. Thus, every rheumatologist or oncologist has to resolve various issues related to the diagnosis, therapy, and monitoring of rheumatic diseases in oncologic patients.

Analyzing the molecular pathways involved in drug-induced carcinogenesis in this disease is also important to identify risk factors that can be minimized. One molecular pathway is associated with increased IL-6 levels and the promotion of WNT signaling. Therefore, tocilizumab, an anti-IL-6 inhibitor, is considered one of the first drugs that can be used if systemic csDMARDs are not efficient in RA patients with lung cancer. In addition, RTX and anti-TNFα are considered to be safe options. It should be noted that further studies are needed on more medications related to the pathways described in this paper, which are beneficial in rheumatic and oncologic diseases.

Furthermore, new studies are needed to establish guidelines for the treatment and planning of screening to prevent cancers, including lung cancer, in RA patients. This is, in part, due to the lack of expert opinion on the pulmonary symptoms of lung cancer and the timeline of screening for cancers in the RA patient population. Future studies should allow the establishment of the rules of medical prevention and intervention in the case of lung cancer development in RA patients using chronically various DMARDs, particularly since, without these drugs, an effective treatment of RA is not possible. Recent EULAR recommendations and our clinical practice prove that DMARDs’ benefits outweigh the potential cancer risks. The intensive studies on new molecule synthesis, which are gradually included in therapeutic programs, should also consider the risk of lung cancer development. Therefore, long-term studies are needed to determine the carcinogenic potential of new biological DMARDs, which are temporarily not included in recommendations for oncologic patients with rheumatic disease due to the short observation period.

Moreover, our paper has several limitations. It has only suggested a strong association between multiple risk factors such as smoking, chronic inflammation, and the possibility of drug-induced carcinogenesis with the incidence of lung cancer in RA rather than causation. Thus, further studies into this subject are strongly encouraged and recommended. The review was also unable to take into account research regarding sulfasalazine and sarilumab as studies were few and irrelevant or did not report the incidence of lung cancer. It would be ideal to study the association of the effects of these commonly used drugs with the incidence of lung cancer in RA patients.

## Data Availability

Data sharing is not applicable.

## Abbreviations

The following abbreviations are used in this manuscript:

ADOR	4 G-protein coupled receptor
aHR	Adjusted hazard ratio
AICAR	5-aminoimidazole-4-carboxamide ribonucleotide
cAMP	Cyclic adenosine monophosphate
cHR	Crude hazard ratio
CI	Confidence interval
CIR	Crude incidence ratio
csDMARDs	Conventional synthetic DMARDs
CQ	Chloroquine
DMARDs	Disease-modifying anti-rheumatic drugs
EAIR	Exposure-adjusted incidence rate
ENT1	Equilibrative nucleoside transporter 1
FAICAR	5-form-aminoimidazole-4-carboxamide ribonucleotide
FOXO	Forkhead box O
NF-kB	Nuclear factor kappa B
GCS	Glucocorticosteroid
GLM	Golimumab
GLM-IV	Golimumab intravenous
HCQ	Hydroxychloroquine
HDL	High-density lipoprotein
HDMTX	Methotrexate is used in high doses
i.v.	Intravenously
IA	Inflammatory arthritis
IFN-γ	Interferon-gamma
IL-1β	Interleukin-1β
IL-6	Interleukin -6
IR	Incidence ratio
JAK	Janus kinase
JAKi	Janus kinase inhibitor
JIA	Juvenile idiopathic arthritis
LDL	Low-density lipoprotein
LEF	Leflunomide
LORA	Late-onset rheumatoid arthritis
miRNA155	Micro RNA 155
MTX	Methotrexate
N/A	Not available or not reported
NF-kB	Nuclear factor kappa B
NSAIDs	Non-steroidal anti-inflammatory arthritis
NSLC	Non-small lung cancer
RA	Rheumatoid arthritis
RCTs	Randomized control trials
RTX	Rituximab
s.c.	Subcutaneously
SCC	Squamous cell carcinoma
SCLC	Small cell lung cancer
SIR	Standardized incidence ratio
SN	Sulfasalazine
SR-B1	Scavenger receptor class B type 1
TAAs	Tumor-associated antigens
TNF-α	Tumor necrosis factor-alpha
tsDMARD	Targeted synthetic DMARD
VEGF	Vascular endothelial growth factor
